# Fine Optimization of Morphology Evolution Kinetics with Binary Additives for Efficient Non‐Fullerene Organic Solar Cells

**DOI:** 10.1002/advs.201801560

**Published:** 2019-01-28

**Authors:** Jianya Chen, Zhaozhao Bi, Xianbin Xu, Qianqian Zhang, Shengchun Yang, Shengwei Guo, Hongping Yan, Wei You, Wei Ma

**Affiliations:** ^1^ State Key Laboratory for Mechanical Behavior of Materials Xi'an Jiaotong University Xi'an 710049 China; ^2^ School of Science MOE Key Laboratory for Non‐equilibrium Synthesis and Modulation of Condensed Matter Xi'an Jiaotong University Xi'an 710049 China; ^3^ Department of Chemistry University of North Carolina at Chapel Hill Chapel Hill NC 27599 USA; ^4^ College of Materials Science and Engineering North Minzu University Yinchuan 750021 China; ^5^ SLAC National Accelerator Laboratory Menlo Park CA 94025 USA; ^6^ State Key Laboratory of Luminescent Materials and Devices South China University of Technology Guangzhou 510640 China

**Keywords:** binary solvent additives, in situ characterization, kinetics, morphology, polymer solar cells

## Abstract

The power conversion efficiency of polymer solar cells (PSCs) is strongly affected by active layer morphology. Here, two solvent additives (ODT: octance‐1,8‐dithiol; DIO: 1,8‐diiodooctane) are used to optimize the bulk heterojunction morphology of FTAZ:ITIC‐Th based PSCs and ≈11% efficiency is obtained, which is 10% higher than the untreated device. Based on the morphological characterizations, the influence of binary solvent additives on manipulating molecular packing and phase separation of blend films is successfully revealed. More importantly, in situ grazing incidence wide‐angle X‐ray scattering characterization is adopted to explore the crucial role played by these two solvent additives at different stages of the film‐forming process, that is, ODT influences the initial stage of the film‐forming process, while DIO later establishes the ultimate photoactive film formation. Due to the impacts of two additives at different film processing stages, an optimal ratio of ODT:DIO (0.375%:0.125%) is obtained, which helps in realizing the optimized morphology.

Solution‐processed polymer solar cells (PSCs) have showed exceeding potential to be one sustainable energy source for their superior advantages of light weight, simple, wearable, and large‐scale roll‐to‐roll processing.^1^, ^2^, ^3^, ^4^, ^5^, ^6^ Continuous research in materials chemistry and persistent work with device engineering in the last couple of decades have yielded the power conversion efficiencies (PCEs) approaching ≈15% by utilizing non‐fullerene‐based bulk heterojunction (BHJ) structure of PSCs.^7^, ^8^, ^9^, ^10^ It is well established that morphology of photoactive layer governs the photovoltaic performance of PSCs.^11^, ^12^, ^13^, ^14^, ^15^, ^16^ By controlling the nanostructures of the organic photoactive blend layer can efficiently generate charges at the interfaces of donor/acceptor interfaces which would transport through their percolation pathways and collected by the respective electrode extraction layers toward maximum solar conversion.^13^, ^17^ Several significant strategies have been adopted for improved interaction of donor/acceptor materials toward favorable phase separation, crystallization, molecular orientation, and interpenetrated network of phases.^18^, ^19^, ^20^, ^21^, ^22^, ^23^ Since the reported use of high boiling point (BP) solvent by Bazan and co‐workers to optimize the thin film morphology,^20^ many studies have shown the significant impact of the selection of solvent and solvent additive. Besides the consideration of solvent, thermal and solvent‐based annealing strategies were also implemented to conceive optimized nanostructures of photoactive layer toward efficient photovoltaic (PV) performance of PSCs.

Host solvent with lower BP and the additive with relatively higher BP have different vaporizing speed—composition and thus the solvency of the solvent mixture will change gradually during film processing. By incorporating a small amount of specific processing additives into the host solvent, it is possible to control the morphology of BHJ. Consequently, morphologies of the BHJ layers would be modified due to the changed composition of solvent mixtures irrespective of the selection of different additives. The effect of solvent additives on morphology control can be attributed to two properties: their selective solvency toward donor or acceptor and their low volatility.^21^, ^22^ The effects of single solvent additive on the performance of PSCs have been reported for both fullerene and non‐fullerene‐based blends.^24^, ^25^, ^26^, ^27^, ^28^, ^29^ However, the morphology optimization with multiple additives and its influence on PV parameters has rarely been reported so far for non‐fullerene‐based blends. Investigating the influence of multiple additives on molecular packing and phase separation of blends at different length scale and time scale would present guidance toward additive selection for morphology control of the photoactive layer of PSCs.

On the other hand, a thorough understanding of the film growth mechanisms toward a favorable morphology in the initial casting is seriously required to scale processing of these materials toward manufacturing highly efficient devices. To analyze the prospect, the in situ grazing incidence wide‐angle X‐ray scattering (GIWAXS) can be adopted for real‐time characterization of the molecular packing.^30^, ^31^ Only a few studies have focused on the systematic in situ characterization of the crystalline morphology of non‐fullerene‐based blends during spin‐coating, because the most of non‐fullerene systems are processed by low boiling point solvent chloroform (CF), which is volatile and too fast to get the structural information during the film‐forming process. At the same time, using of solvent additive is a common method to optimize the solid‐state morphology, and the central theme of this method is optimizing film dynamic process. There is some understanding of this process for fullerene systems,^23^, ^32^, ^33^ however, dynamic processes involved in low boiling point CF and the effects of additives in filming process are still unclear for non‐fullerenes systems. Understanding series of deep‐seated basics of dynamic processes involved in CF and additives during the film‐forming process would be of great importance, guidance for the morphology control toward photovoltaic performance optimization.

In this work, a binary additives system consisting of ODT and DIO was used to fine tuning the morphology of the active layer processed from the solution of FTAZ:ITIC‐Th (1:1.5, w/w) in CF (the chemical structures are shown in **Scheme** **1**). Interestingly, the ratio of ODT and DIO of the binary additives system strongly affect the PV parameters of the devices, especially *J*_sc_ and FF. When a binary additives system of ODT:DIO (0.375%:0.125%) was used, a champion PCE of 10.93% together with a *V*_oc_ of 0.922 V, a *J*_sc_ of 17.78 mA cm^−2^, and a FF of 66.64% was achieved, which is 23% higher than the previous reported PCE (8.88%). The in situ GIWAXS was implemented to gain insight into the evolution of the active layer morphology during the film‐forming process. It was found that ODT works at the initial stage while DIO works preferentially at a longer time scale.

**Scheme 1:**
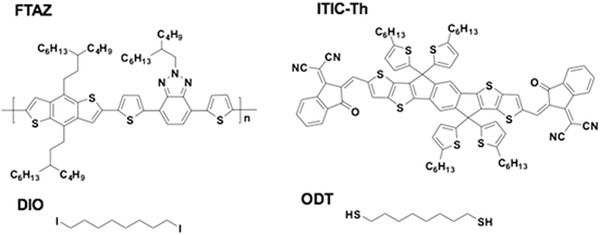
Molecular structures of FTAZ, ITIC‐Th, DIO, and ODT.

According to the literature,^34^ an optimized D/A ratio of 1:1.5 was adopted for the following photovoltaic characterization. PSC devices with an inverted structure of ITO/ZnO/FTAZ:ITIC‐Th (1:1.5, w/w)/MoO_3_/Ag were fabricated. The active layers of the devices were prepared by spin‐casting the FTAZ:ITIC‐Th solutions in CF with ODT (BP ≈ 270 °C) and DIO (BP ≈ 330 °C) as solvent additives. Excepting from the using of the additive, no extra treatment was adopted. Detailed device data are shown in **Table** **1** and Figure S1b (Supporting Information). The devices based on the as‐cast blend film casted from CF show a PCE of 9.53% with a *V*_oc_ of 0.907 V, a *J*_sc_ of 16.8 mA cm^−2^, and a FF of 62.57%. After addition of 0.5% DIO, the *V*_oc_ and *J*_sc_ slightly decreased to 0.879 V and 16.4 mA cm^−2^ while the FF maintained and consequently the PCE decreased to 9.04%, indicating that the content of DIO is excessive when it is 0.5%. On the other hand, when 0.5% ODT was used as solvent additive, the PCE of the devices significantly increased to 10.44%, which is mainly attributed to the greatly enhanced *J*_sc_ and FF. Further increasing the volume of ODT over 0.5% causes the decrease of PCE (Figure S1a and Table S1, Supporting Information). However, the devices treated with the combination of 0.5% DIO and 0.5% ODT showed almost identical photovoltaic parameters to that of the devices with no additives, which indicates that the addition of ODT counteracts the negative effect of DIO. Therefore, the total volume of these two additives was kept at 0.5% to further optimize the performance of devices by gradually increasing the content of DIO from 0.125 to 0.25% and to 0.375%. Eventually, the devices treated with binary additives system consist of 0.375% ODT and 0.125% DIO gave out the highest PCE of 10.93% with enhancement of *V*_oc_, *J*_sc_, and FF simultaneously (**Figure** **1**a). The absorption spectra of blend films processed with different additive ratios are plotted in Figure S2 (Supporting Information). The charge carrier motilities of the active layers processed with different amount of additive were measured by the space‐charge limited current (SCLC) method (Figure S3, Supporting Information). The hole mobility was calculated to be 8.42 × 10^−4^, 9.30 × 10^−4^, and 8.51 × 10^−4^ cm^2^ V^−1^ s^−1^ for active layers treated without additive and with binary additives of ODT:DIO (0.375%:0.125%) and ODT:DIO (0.5%:0.5%), respectively. The electron mobility was measured to be 1 × 10^−5^, 1.67 × 10^−4^, and 7.91 × 10^−5^ cm^2^ V^−1^ s^−1^ for active layers treated without additive and with binary additives of ODT:DIO (0.375%:0.125%) and ODT:DIO (0.5%:0.5%), respectively. The greatly enhanced electron mobility for the active layers treated with the ODT:DIO (0.375%:0.125%) improved the charge carrier mobility balance, which may reduce the charge carrier recombination and leading to the highest *J*_sc_ and FF (shown in Figure S4, Supporting Information). As seen from the EQE curves (Figure 1b), the binary additives system of ODT:DIO (0.375%:0.125%) greatly improved the optical response of the devices from 400 to 700 nm with EQE reach 80% from 520 to 720 nm, which correlates well with the enhanced *J*_sc_ compared to the devices without additives and with ODT:DIO (0.5%:0.5%). The lower EQE values across almost the entire EQE spectra for the devices treated with ODT:DIO (0.5%:0.5%) also correspond well with their lower *J*_sc_. The integrated current density of the EQE spectra were 15.9, 17.41, and 16.6 mA cm^−2^ for the devices treated without additives, and with ODT: DIO (0.375%:0.125%) and ODT:DIO (0.5%:0.5%), respectively. The deviations between the integral current densities and the *J*_sc_ read from *J–V* measurements are all within ±3.9%, indicating the good consistency of the photovoltaic results. Due to the effective method of binary additives, devices based on PTB7‐Th^35^:ITIC^36^ and PTB7‐Th:ITIC‐Th^37^ were also made for expanding this method to other BHJ systems, the photovoltaic performances are shown in Figure S5, Tables S2 and S3 (Supporting Information). The PCEs of these system devices with binary additives both increased, indicating an extensible method for high‐performance OSCs. Notably, the changes of *V*_oc_ with different additive ratios show interesting effects of binary additives on BHJs and deserve deeper investigation, which is out of the scope of this paper.

**Table 1:** Photovoltaic performance of the devices based on FTAZ:ITIC‐Th (1:1.5, w/w) without or with various ratios of two additives under the illumination of AM 1.5G, 100 mW cm^−2^

ODT:DIO	*V*_OC_ [V]	*J*_sc_ [mA cm^−2^]	FF [%]	PCEa) [%]
No additive	0.907(±0.003)	16.8(±0.23)	62.57(±0.73)	9.53(±0.19)
0:0.5%	0.879(±0.007)	16.4(±0.08)	62.69(±0.6)	9.04(±0.36)
0.5%:0.5%	0.918(±0.002)	16.55(±0.25)	62.58(±1.64)	9.51(±0.25)
0.5%:0	0.884(±0.003)	17.82(±0.21)	66.3(±0.36)	10.44(±0.19)
0.375%:0.125%	0.922(±0.004)	17.78(±0.28)	66.64(±0.47)	10.93(±0.22)
0.25%:0.25%	0.872(±0.005)	17.48(±0.3)	66.17(±0.2)	10.1(±0.24)
0.125%:0.375%	0.876(±0.004)	16.71(±0.4)	64.39(±0.32)	9.42(±0.3)

^a)^ Average values calculated from 20 devices.

**Figure 1:**
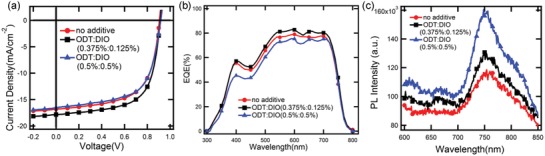
a) *J*–*V* curves and b) EQE of the PSCs under the illumination of AM 1.5G, 100 mW cm^−2^, c) photoluminescence spectra of blend films processed without additive and with ratios of ODT:DIO (0.375%:0.125%, 0.5%:0.5%).

We have also investigated the photoluminescence (PL) intensities of blend films processed without and with additives of ODT:DIO (0.375%:0125%, 0.5%:0.5%) (Figure 1c). For the as‐cast blend, the PL intensity is the lowest, which indicating an efficient charge dissociation. For the blend processed with ODT:DIO 0.5%:0.5%, the PL is the highest, suggesting a less efficient charge dissociation.^38^ The PL intensity of the optimized blend is in‐between the two. The PL intensity, that is, charge dissociation efficiency, strongly correlates with the morphology of active layers. Hence, we carried out the detailed morphology characterization in next section.

It is well established that the performance of PSCs is closely related to the film morphology. Various structural aspects of phase‐separated domains in BHJ can be quantified with resonant soft X‐ray scattering (R‐SoXS), including domain size and relative composition variation.^39^, ^40^ Photon energy of 285.8 eV was selected to provide highly enhanced materials contrast. The R‐SoXS profiles of different blend films processed with various ratios of binary additives are shown in **Figure** **2**. The average composition variation (relative domain purity) is revealed via integrating the scattering profile and calculating the total scattering intensity (TSI).^41^ For the blend film without additive, a scattering peak at *q* ≈ 0.302 nm^−1^ arises, which corresponds to a small domain size of 10 nm. Meanwhile, the relative domain purity of this blend film is calculated to be 71%. It is well known that a small and impure phase would hinder the charge transport.^13^ The domain size and relative domain purity for the blend film treated with 0.5% DIO and 0.5% ODT are calculated to be 71 nm and 16 nm, and 66% and 74%, respectively. When the binary additives ODT:DIO (0.5%:0.5%) are used, two scattering peaks located at *q* ≈ 0.017 and 0.13 nm^−1^ arise, corresponding to the domain size of 180 and 24 nm, respectively. Obviously, the large domain size and low relative domain purity of the 0.5% DIO‐treated blend films will definitely hinder the charge separation and transport, leading to their inferior PV performance. For the ODT:DIO (0.5%:0.5%) treated devices, despite their highest relative domain purity (100%), the extensively large domain size of 184 nm is fatal for achieving high PV performance. However, because of the existence of some smaller domains (24 nm), their PV parameters still outperform those of the devices with only 0.5% DIO. As for the 0.5% ODT‐treated devices, the suitable domain size of 16 nm and high relatively domain purity of 74% benefit the charge separation and transport, leading to their superior PV performance with an average PCE of 10.44%. Furthermore, the domain size of active layers for the binary additives system with different ratios of ODT:DIO (0.375%:0.125%, 0.25%:0.25%, and 0.125%:0.375%) is calculated to be 14, 12, and 15 nm, respectively. With the increasing content of DIO of binary additives, the relative domain purity for three blend films is almost same (79, 76, and 83%, respectively). Compared with the blend films processed with 0.5% DIO, 0.5% ODT, and ODT:DIO (0.5%:0.5%), an appropriate phase separation with domain size within 10–20 nm can be achieved with the binary additives treatment, which is favorable for effective charge dissociation. Even though the relative domain purity of the ODT:DIO (0.375%:0.125%) is not the highest among the binary additives processed devices, the synergistic effect of more balanced charge carrier mobility (Figure S2, Supporting Information), appropriate domain size and relative purity helped boosting their PV performance to a PCE of 10.93%. Transmission electron microscopy (TEM) scan was conducted to gain intuitive observation of active layer morphology (Figure S6, Supporting Information), and found them consistent with our observations in R‐SoXS data. As shown in Figure S6 (Supporting Information), the donor and acceptor mixed evenly when the blend processed without additive, and form small and impure phase. In contrast, the blend with the additive ratio of ODT:DIO ≈ 0.375%:0.125% shows small and phase. Larger domains at the length scale of 200 nm were observed when the film is fabricated with additive of ODT:DIO 0.5%:0.5%.

**Figure 2:**
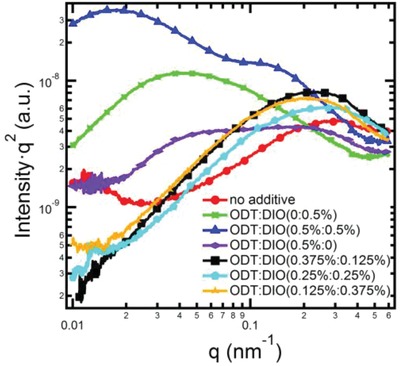
R‐SoXS data reduced from 2D detector images for blend films processed without and with various ratio of ODT and DIO.

To investigate the polymer crystallinity and lamellar spacing, grazing incident wide‐angle X‐ray scattering (GIWAXS) was conducted to provide the structural information including crystallite size, intermolecular distance, and crystallite orientation in blend films. **Figure** **3** shows the GIWAXS 2D patterns and line profiles of the blend films without additive and with different ODT:DIO ratios. For all of the investigated films, pronounced (010) π–π stacking peaks in the out‐of‐plane direction can be seen with no marked (010) π–π stacking peaks in the in‐plane direction, indicating the preferential face‐on orientation with respective to the substrate. The line profiles of the FTAZ and ITIC‐Th pure films (Figure S7, Supporting Information) are used to define the attribution of peaks in the binary blend films. It is clear that the scattering profile of FTAZ:ITIC‐Th without additive is very similar to that of the FTAZ pure film, which indicates that the ITIC‐Th has little impact on crystallization of FTAZ when processed without additive and the diffraction of ITIC‐Th will dominate in the blend when additives are added. Gaussian fitting was conducted to the (010) π–π stacking peaks of the blend films and the coherence lengths (CL) of (010) π–π stacking peaks (Table S4, Supporting Information) are calculated using the Scherrer equation. As shown in Figure 3, the crystallinity of the system was significantly improved when 0.5% DIO was added, with the CL increased from 30 to 34 Å. The crystallinity was further increased when an additional 0.5% ODT was added. Nevertheless, with only 0.5% ODT used as additive, the crystallinity was enhanced compared to that when no additives were used, but effect was not as pronounced as either in the cases of 0.5% DIO or 0.5% ODT:0.5% DIO. Interestingly, the morphology of blend films processed with binary additives (at the range of 0–0.5%) is very similar to that of the films processed with only 0.5% ODT. The reason for this difference in this small range is most likely dominated by ODT during film‐forming processes, which will be discussed in next section in detail. Among the three BHJ films processed with binary additives, the blend film processed with ODT:DIO (0.25%:0.25%) showed the lowest CL, which is the proof of the counteracting behavior of two additives as we observed in device performance. Combining the quantitative information of domain sizes and relative domain purity from R‐SoXS, and degree of crystallization from GIWAXS characterization, the contradiction between PL and photovoltaic parameters can be rationalized. It is widely accepted that a small and impure phase is good for charge separation and thus low PL intensity (with additive), while a large and purer phase poorly contributes to the charge separation (0.5%:0.5% additives) and thus high PL intensity. The domain size and domain purity of the blend processed with ODT:DIO (0.375%:0.125%) is between the first two, so the result of PL has such a trend (Figure 1c).

**Figure 3:**
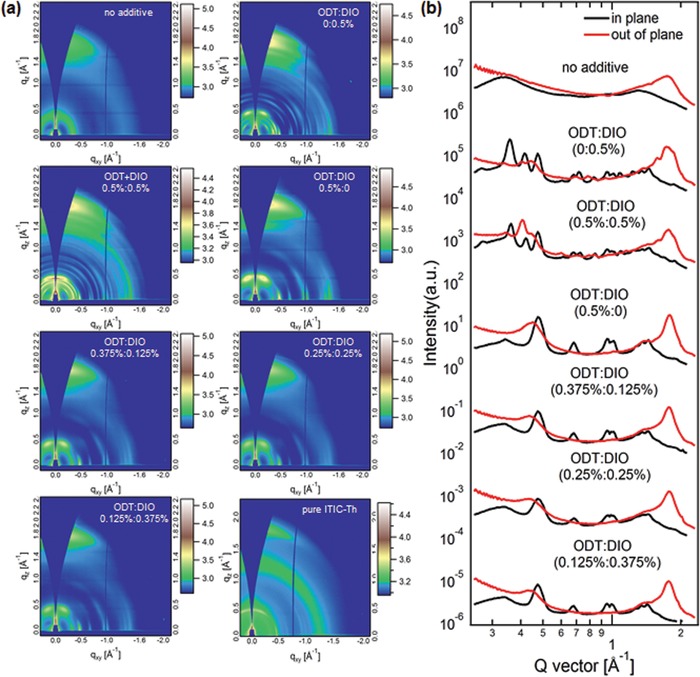
a) GIWAXS 2D scattering patterns and b) line profiles of FTAZ/ITIC‐Th blend films processed without and with different ratios of additive.

In order to better understand the variation of molecular packing as a function of the ratio of two additives in solid thin film. The in situ GIWAXS characterization was carried out to probe the dynamic process of morphology evolution for the blend films during spin‐coating. **Figure** **4**a shows the data collection diagram. False‐color 2D images (Figure 4b) were produced to help better view the evolution of the scattering profiles in out‐of‐plane direction, with the horizontal axis being the scattering vector *q*, vertical being the elapsed time with respect to the onset of sample rotating during spin‐coating, and color being the scattering intensity. A summary of the area and the *d*‐spacing of (010) π–π stacking diffraction for the blend with ratios of ODT:DIO (0.5%:0, 0.375%:0.125%, 0:0.5%) is shown in Figure 4c. In Figure 4b, the most significant diffraction is the peak with *q* = 1.79 Å^−1^ (indicated by an arrow labeled “π–π stacking”). As mentioned in the previous section with the ex situ GIWAXS characterization, this diffraction peak is mainly originated from ITIC‐Th and thus we analyze in detail the variation of this peak as a function of the time. The area of diffraction peak is extracted from Gaussian fitting, and it is the indicative of the crystallinity of films. The area of π–π stacking peak for ITIC‐Th in blend films processed with 0.5% ODT hardly changes after 100 s while that of the 0.5% DIO processed films gradually shifts to high *q* direction (1.79 Å^−1^) till almost 600 s. This indicates that the low BP ODT functions at the beginning stage of the film forming while the high BP DIO works dominantly throughout the whole film‐forming process. Based on this observation, we conclude that the ODT vaporizes faster than the DIO and thus ODT controls the initial stage of the film forming and the DIO controls the later stage of the film forming. For the binary additives (ODT:DIO 0.375%:0.125), the area of π–π stacking peak is in between the pure ODT and DIO as shown in Figure 4c. ODT plays a main role at the beginning of the film (<100 s) and DIO works in the later filming process (>100 s). We further note that, for the optimal conditions ODT:DIO 0.375%:0.125%, the amount of ODT is greater than the amount of DIO and the area of diffraction peak stabilized at near 400 s. Comparison of the effects of using binary additives might suggest that the major impact of ODT in the drying process is promoting the nucleation. Because of the relatively low boiling point comparing with DIO, the ODT evaporates relatively faster and the crystallization of the film stops and the area does not grow over time after 100 s. In contrast, DIO has higher boiling point, it stays in the film for longer time, serving as plasticizer and allowing longer time for molecule rearrangement to promote big crystalline domains. This analysis explains why the molecular packing is weak with equal ODT:DIO ratio 0.25%:0.25%. Due to the lack of enough nucleation agent with 0.25% ODT and the weak driving force for crystallization and molecular reorganization with 0.25% DIO, and thus finally the molecular packing is weak.

**Figure 4:**
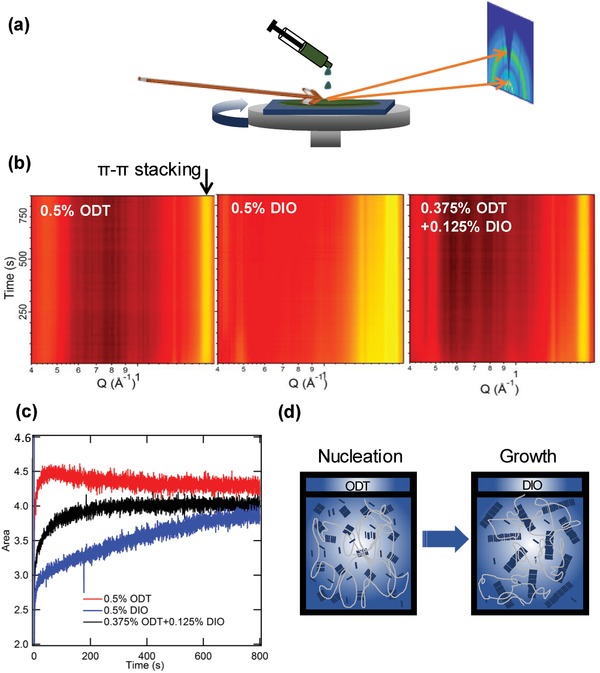
a) The collection diagram of in situ GIWAXS data, b) 2D representation images of morphology evolution for blend films with ODT, DIO, and ODT:DIO (0.375%:0.125%) binary additives. c) Evolution of the fitted (010) diffraction peak area of these films obtained under different processing conditions. d) Schematic diagram of the effects of two additives on the crystallinity of the material.

In conclusion, a binary additive consisting of ODT and DIO was used to fine optimize the PV performance of FTAZ:ITIC‐Th. The morphologies of blend films processed without or with different ratio of two additives were systematically investigated by GIWAXS, R‐SoXS, TEM, and in situ GIWAXS. The GIWAXS and R‐SoXS data reveal that the *J*_sc_ and FF are strongly influenced by the domain sizes and the relative domain purity. With the ODT:DIO (0.375%:0.125%) treatment, an optimal morphology with appropriate domain size and relative domain purity could be achieved, leading to a high PCE of 10.93% together with a *V*_oc_ of 0.922 V, a *J*_sc_ of 17.78 mA cm^−2^, and a FF of 66.64%. In situ GIWAXS was performed to gain insight into the film‐forming kinetics processed with the binary additives. It was found that ODT preferentially works at the beginning stage of the film forming to control nucleation of the crystal while the DIO's effect on the film forming last throughout the whole film‐forming process to control the crystal growth. This work provides informative and useful guidance to solvent additive selection for morphology control of PSCs.

## Conflict of Interest

The authors declare no conflict of interest.

## Supporting information

Supplementary
