# Supplementary material for: Fine Optimization of Morphology Evolution Kinetics with Binary Additives for Efficient Non‐Fullerene Organic Solar Cells
Source: Adv Sci (Weinh). 2019 Jan 28;6(6):1801560. doi: 10.1002/advs.201801560 (PMC6425445; doi:10.1002/advs.201801560)
Supplement: Supplementary file 1 — Supplementary [file ADVS-6-1801560-s001.pdf]

Copyright WILEY-VCH Verlag GmbH & Co. KGaA, 69469 Weinheim, Germany, 2018.

# Supporting Information

for *Adv. Sci.,* DOI: 10.1002/advs.201801560

# Fine Optimization of Morphology Evolution Kinetics with Binary Additives for Efficient Non-Fullerene Organic Solar Cells

*Jianya Chen, Zhaozhao Bi, Xianbin Xu, Qianqian Zhang, Shengchun Yang,* Shengwei Guo,* Hongping Yan, Wei You, and Wei Ma**

Copyright WILEY-VCH Verlag GmbH & Co. KGaA, 69469 Weinheim, Germany, 2018.

# Supporting Information

**Title: Fine Optimization of Morphology Evolution Kinetics with Binary additives for Efficient Non-Fullerene Organic Solar Cells**

Jianya Chen#, Zhaozhao Bi#, Xianbin Xu, Qianqian Zhang, Shengchun Yang*, Shengwei Guo*, Hongping Yan, Wei You, Wei Ma*

## Experimental Section

### Materials

ITIC-Th was purchased from Solarmer Materials Inc. and used as received. FTAZ was provided by Prof. W. You. Two solvent additives and the solvents used in device fabrication process were purchased from Alfa Aesar. Commercial available chemicals are used without further purification.

### Device Fabrication:

PSCs were fabricated with an inverted structure of ITO/ZnO/active layer/MoOx/Ag. ITO glass (sheet resistance = 15 Ω $sq^{-1}$) was precleaned in an ultrasonic bath with acetone and isopropanol, and treated in an ultraviolet ozone chamber for 20 min. ZnO electron transport layers were prepared onto the ITO glass through spin coating at 4500 rpm from a ZnO precursor solution. Then, the ZnO substrates were immediately baked in air at 200 °C for 30 min. A chloroform solution (totally 12 mg $mL^{-1}$) FTAZ: ITIC-Th was subsequently spin-coated at 5000 rpm on the ZnO layer to form a photosensitive layer (*ca.* 80-100 nm). The thickness of the photosensitive layer was measured using an Ambios Technology $XP^{-2}$ profilometer. Finally, thin layer (10 nm) of MoOx was deposited as the anode interlayer, and

80 nm of Ag was deposited as the top electrode under vacuum (*ca.* $10^{-5}$ Pa). The active area of the device was *ca.* 4 $mm^2$. The *J-V* curves were measured using a Keithley 2400 source-measure unit under ambient condition. Photocurrent was measured under AM 1.5G illumination at 100 mW $cm^{-2}$ using a AAA solar simulator (SS-F5-3A, Enli Technology CO., Ltd.) calibrated with a stander photovoltaic cell equipped with a KG5 filter. The EQE measurements of the devices were carried out in the air via using a solar cell spectral response measurement system (QE-R3018, Enli Technology CO. Ltd) with the calibrated light by a standard single-crystal Si photovoltaic cell.

**Mobility measurements.**

Hole- and electron-only diodes were fabricated using the architectures: ITO/PEDOT:PSS/active layer/MoOx/Ag for holes and ITO/ZnO/active layer/Ca/Al for electrons. Mobilities were extracted by fitting the current density–voltage curves using space charge limited current (SCLC). The *J–V* curves of the devices were plotted as $J^{1/2}$ versus $V$ using Eq. $J = 9\varepsilon_0\varepsilon_r\mu_h(\mu_e)V^2/8d^3$ for holes and electrons, where $J$ is current density, $d$ is film thickness of active layer (80 to 100 nm), $\mu_h$ is hole mobility, $\mu_e$ is electron mobility, $\varepsilon_r$ is relative dielectric constant of the transport medium, and $\varepsilon_0$ is permittivity of free space ($8.85 \times 10^{-12}$ $Fm^{-1}$). $V = V_{appl}–V_{bi}$, $V_{appl}$ is the applied voltage, $V_{bi}$ is the offset voltage.

**TEM characterization**

The transmission electron microscopy (TEM) characterization was carried out on JEM-1011. The samples for the TEM measurements were prepared as follows: the active-layer films were spin-cast onto ITO/PEDOT:PSS substrates, and the substrates with the active layers were submerged in deionized water to make the active layers float on the air–water interface. Then, the floated films were picked up on unsupported 200 mesh copper grids for the TEM measurements.

**Grazing Incidence Wide-Angle X-ray Scattering (GIWAXS) Characterization**:

GIWAXS measurements were performed at beamline 7.3.3[1] at the Advanced Light Source. Samples were prepared on Si substrates using identical blend solutions as those used in devices. The 10 keV X-ray beam was incident at a grazing angle of 0.12° - 0.15°, selected to maximize the scattering intensity from the samples. The scattered x-rays were detected using a Dectris Pilatus 2M photon counting detector.

**Resonant Soft X-ray Scattering (RSoXS) Characterization**:

RSoXS transmission measurements were performed at beamline 11.0.1.2[2] at the Advanced Light Source (ALS). Samples for R-SoXS measurements were prepared on a PSS modified Si substrate under the same conditions as those used for device fabrication, and then transferred by floating in water to a 1.5 mm × 1.5 mm, 100 nm thick $Si_3N_4$ membrane supported by a 5 mm × 5 mm, 200 μm thick Si frame (Norcada Inc.). 2-D scattering patterns were collected on an in-vacuum CCD camera (Princeton Instrument PI-MTE). The sample detector distance was calibrated from diffraction peaks of a triblock copolymer poly (isoprene-b-styrene-b-2-vinyl pyridine), which has a known spacing of 391 Å. The beam size at the sample is approximately 100 μm by 200 μm.

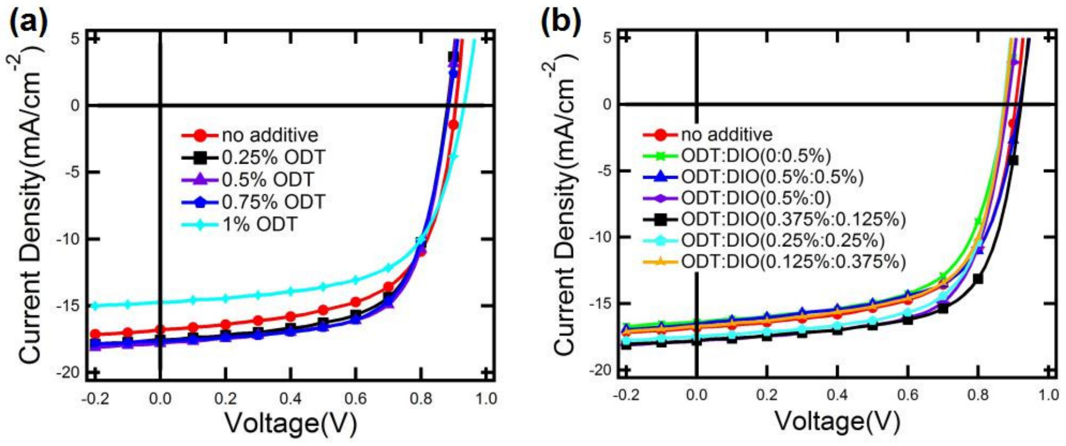

**Figure S1.** *J-V* curves of the PSCs based on FTAZ/ITIC-Th(1:1.5, w/w) with different content of ODT (a) and the PSCs with various ratios of additives (b) under illumination of AM1.5G, 100 mW $cm^{-2}$

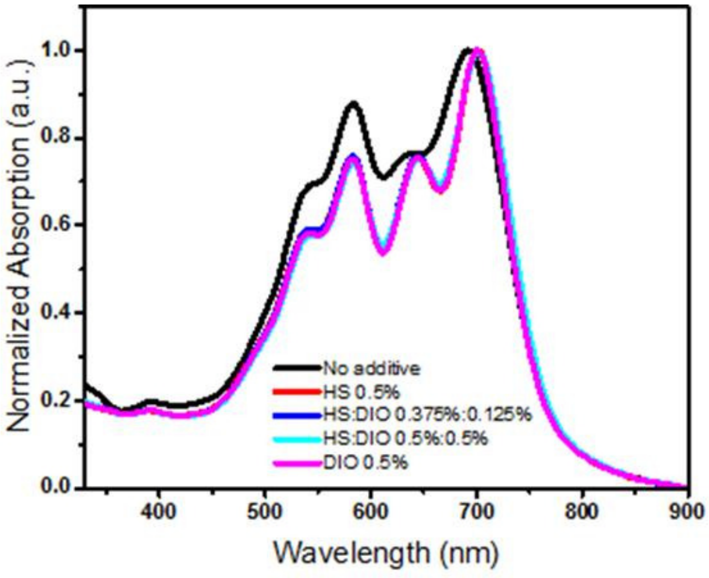


**Figure S2**. Absorption spectra of the blend films processed with different additive ratios

**Table S1.** Photovoltaic performance of the devices based on FTAZ/ITIC-Th (1:1.5, w/w) with different content of ODT under the illumination of AM1.5G, 100 mW $cm^{-2}$

| Additive(ODT) | Voc(V) | Jsc(mW $cm^{-2}$) | FF(%) | PCE(%) |
|---|---|---|---|---|
| No additive | 0.907(±0.003) | 16.8(±0.23) | 62.57(±0.73) | 9.53(±0.19) |
| 0.25% | 0.883(±0.005) | 17.57(±0.18) | 64.98(±0.8) | 10.07(±0.2) |
| 0.5% | 0.884(±0.003) | 17.82(±0.21) | 66.3(±0.6) | 10.44(±0.19) |
| 0.75% | 0.886(±0.007) | 17.67(±0.15) | 65.48(±0.63) | 10.25(±0.2) |
| 1% | 0.931(±0.006) | 14.47(±0.2) | 62.22(±0.4) | 8.56(±0.06) |

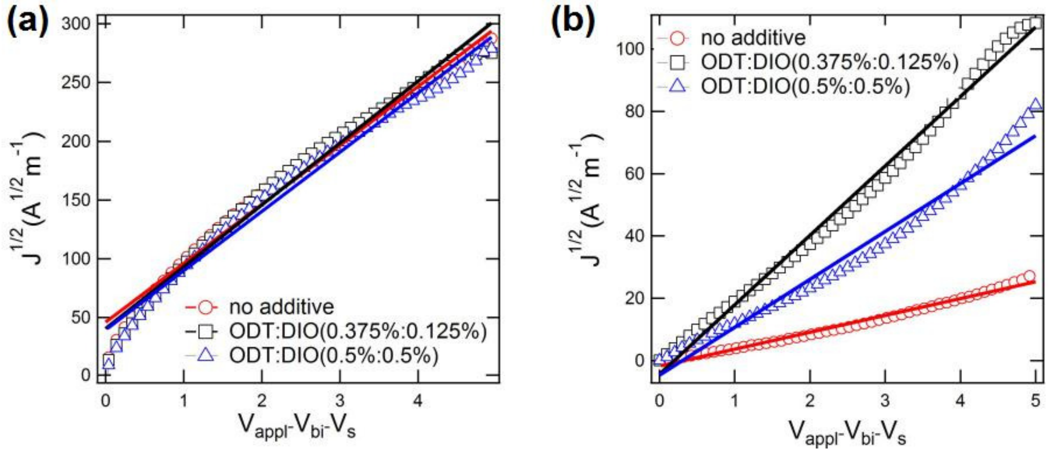


**Figure S3.** $J^{1/2}$–$V$ plots for FTAZ:ITIC-Th (1:1.5, w/w)-based hole-only devices (a) electron-only devices (b).

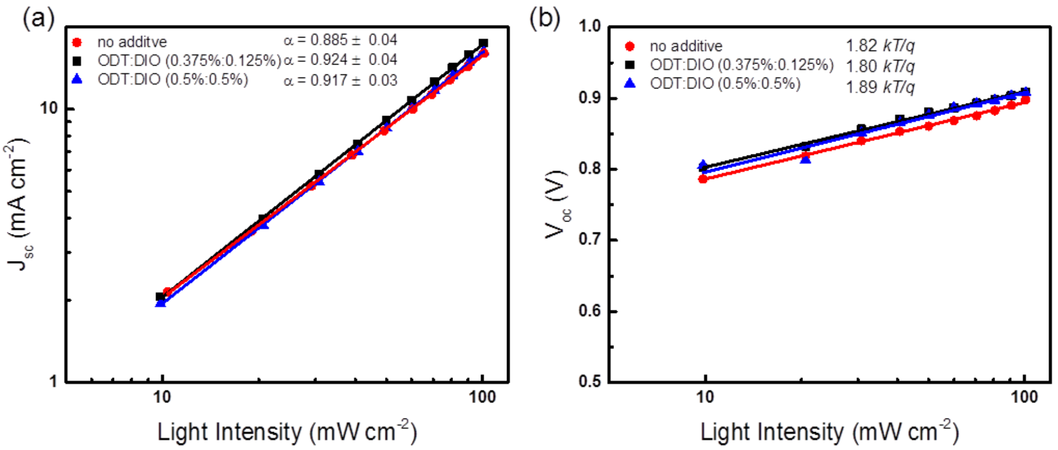


**Figure S4.** (a) Dependence of $J_{sc}$ on light intensity; (b) dependence of $V_{oc}$ on light intensity for OSCs devices processed with and without additives.

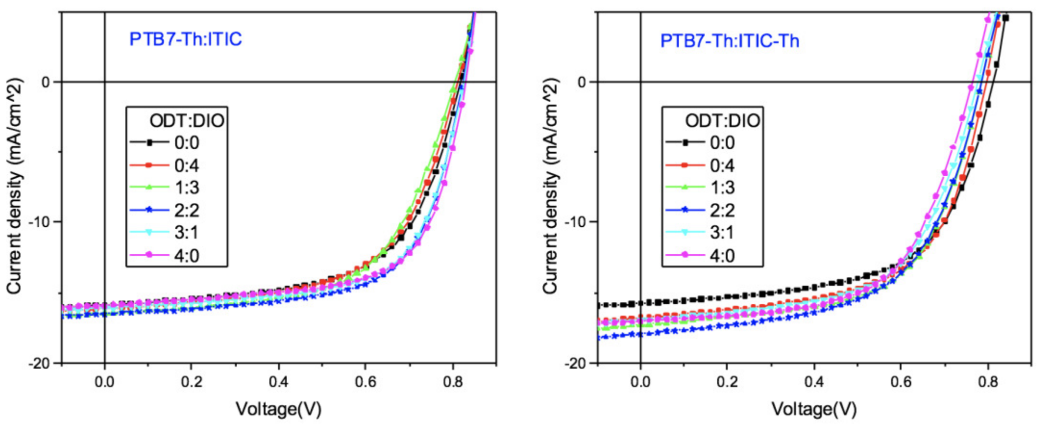

**Figure S5**. J-V curves of PTB7-Th:ITIC devices and PTB7-Th:ITIC-Th devices with different additive ratios under the illumination of AM 1.5G, 100 mW $cm^{-2}$.

**Table S2.** Photovoltaic properties of PTB7-Th:ITIC devices with different additive ratios under illumination of AM 1.5G, 100 mW $cm^{-2}$.

| ODT:DIO | Voc (V) | Jsc (mA $cm^{-2}$) | Fill Factor (%) | Efficiency (%) |
|---|---|---|---|---|
| 0 | 0.817±0.003 | 15.91±0.27 | 60.63±0.35 | 7.92±0.15 |
| 0:4 | 0.811±0.004 | 16.12±0.29 | 59.64±0.22 | 7.83±0.22 |
| 1:3 | 0.805±0.003 | 16.48±0.33 | 59.74±0.26 | 7.97±0.20 |
| 2:2 | 0.822±0.004 | 16.51±0.31 | 64.74±0.42 | 8.83±0.32 |
| 3:1 | 0.824±0.002 | 16.08±0.30 | 64.94±0.49 | 8.65±0.22 |
| 4:0 | 0.828±0.004 | 15.92±0.28 | 65.72±0.45 | 8.65±0.27 |

**Table S3.** Photovoltaic properties of PTB7-Th:ITIC-Th devices with different additive ratios under illumination of AM 1.5G, 100 mW $cm^{-2}$.

| ODT:DIO | Voc (V) | Jsc (mA $cm^{-2}$) | Fill Factor (%) | Efficiency (%) |
|---|---|---|---|---|
| 0 | 0.811±0.003 | 15.75±0.13 | 60.23±0.35 | 7.71±0.19 |
| 0:4 | 0.795±0.002 | 16.78±0.58 | 60.08±0.12 | 8.03±0.31 |
| 1:3 | 0.786±0.003 | 17.32±0.37 | 60.29±0.54 | 8.22±0.20 |
| 2:2 | 0.785±0.004 | 17.95±0.32 | 58.11±0.12 | 8.20±0.11 |
| 3:1 | 0.777±0.007 | 16.95±0.13 | 59.26±0.57 | 7.82±0.14 |
| 4:0 | 0.764±0.012 | 17.03±0.45 | 60.12±0.87 | 7.83±0.43 |

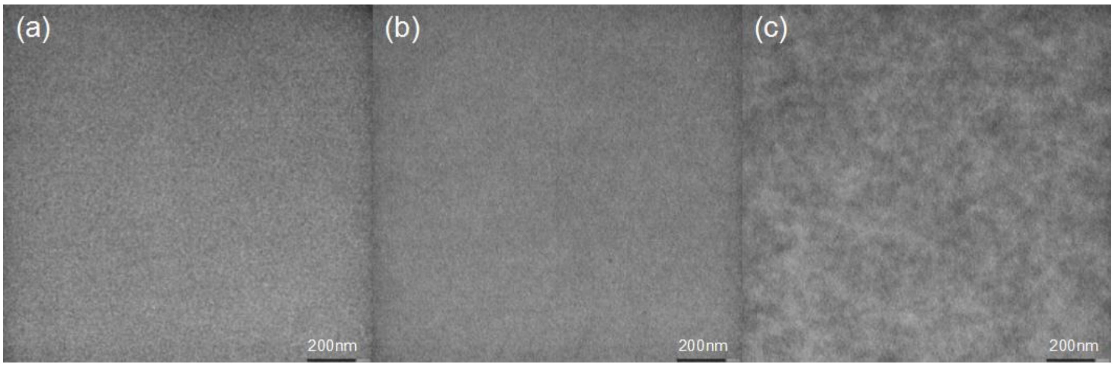


**Figure S6.** TEM images of FTAZ/ITIC-Th blend films without (a) and with different ratios of ODT:DIO ((b) 0.375%:0.125%, (c) 0.5%:0.5%).

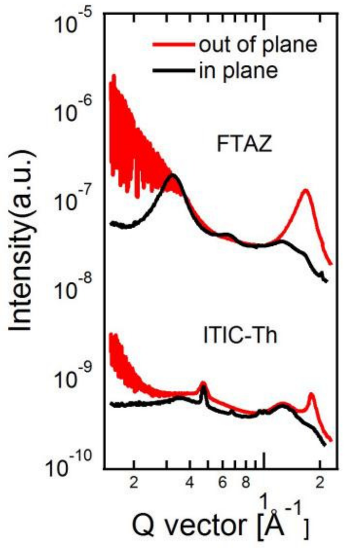


**Figure S7**. GIWAXS scattering line profiles of FTAZ pure film and ITIC-Th pure film processed without additive.

**Table S4.** The fitted data of π–π stacking peaks of blend films processed with various ratios of additives.

| ODT:DIO | FWHM($Å^{-1}$) | CL(Å) |
|---|---|---|
| No additive | 0.188 | 30.04 |
| 0:0.5% | 0.146 | 34.97 |
| 0.5%:0.5% | 0.162 | 38.57 |
| 0.5%:0 | 0.147 | 37.36 |
| 0.375%:0.125% | 0.151 | 40.15 |
| 0.25%:0.25% | 0.141 | 37.00 |
| 0.125%:0.375% | 0.151 | 38.69 |

**Reference**

[1] A. Hexemer, W. Bras, J. Glossinger, E. Schaible, E. Gann, R. Kirian, A. MacDowell, M. Church, B. Rude, H. Padmore, *Journal of Physics: Conference Series* **2010**, *247*, 012007.

[2] E. Gann, A. T. Young, B. A. Collins, H. Yan, J. Nasiatka, H. A. Padmore, H. Ade, A. Hexemer, C. Wang, *Rev. Sci. Instrum.* **2012**, *83*, 045110.
